# The Non-Coding RNAs Inducing Drug Resistance in Ovarian Cancer: A New Perspective for Understanding Drug Resistance

**DOI:** 10.3389/fonc.2021.742149

**Published:** 2021-09-30

**Authors:** Gaofeng Li, Jun Gong, Shulong Cao, Zhaoyang Wu, Dong Cheng, Jia Zhu, Xuqun Huang, Jingyi Tang, Yuning Yuan, Wenqi Cai, Haiyuan Zhang

**Affiliations:** ^1^ School of Basic Medicine, Health Science Center, Yangtze University, Jingzhou, China; ^2^ Department of Abdominal and Pelvic Medical Oncology, Huangshi Central Hospital, Affiliated Hospital of Hubei Polytechnic University, Edong Healthcare Group, Huangshi, China; ^3^ Hubei Cancer Hospital, Tongji Medical College, Huazhong University of Science and Technology, Wuhan, China; ^4^ Hubei Enshi College, Enshi, China; ^5^ Department of Thoracic Medical Oncology, Huangshi Central Hospital, Affiliated Hospital of Hubei Polytechnic University, Edong Healthcare Group, Huangshi, China

**Keywords:** non-coding RNAs, ovarian cancer, chemotherapy, drug resistance (DR), cisplatin

## Abstract

Ovarian cancer, a common malignant tumor, is one of the primary causes of cancer-related deaths in women. Systemic chemotherapy with platinum-based compounds or taxanes is the first-line treatment for ovarian cancer. However, resistance to these chemotherapeutic drugs worsens the prognosis. The underlying mechanism of chemotherapeutic resistance in ovarian cancer remains unclear. Non-coding RNAs, including long non-coding RNAs, microRNAs, and circular RNAs, have been implicated in the development of drug resistance. Abnormally expressed non-coding RNAs can promote ovarian cancer resistance by inducing apoptosis inhibition, protective autophagy, abnormal tumor cell proliferation, epithelial-mesenchymal transition, abnormal glycolysis, drug efflux, and cancer cell stemness. This review summarizes the role of non-coding RNAs in the development of chemotherapeutic resistance in ovarian cancer, including their mechanisms, targets, and potential signaling pathways. This will facilitate the development of novel chemotherapeutic agents that can target these non-coding RNAs and improve ovarian cancer treatment.

## Introduction

Ovarian cancer is a relatively common malignant tumor in women that easily metastasizes in its advanced stages (1–3). Systemic chemotherapy remains the mainstay treatment of advanced ovarian cancer (4, 5). However, the emergence of drug resistance has limited its clinical application (6).

Ovarian cancer cells have developed multiple resistance mechanisms, including impairment in cellular copper transporters, intracellular detoxification, DNA damage repair (DDR), and non-coding RNA (ncRNA)-mediated drug resistance. Copper transporter 1 (CTR1), ATP7A, and ATP7B participate in the uptake or efflux of platinum (7, 8). Interestingly, when their expression is dysregulated, drug concentration within ovarian cancer cells decrease, resulting in drug resistance. Glutathione S-transferase π (GST-π) is an intracellular detoxification enzyme that promotes the conjugation of glutathione (GSH) with chemotherapeutic drugs, and such conjugated drugs are easily excreted and their toxic effects are eliminated; overall, this process also results in drug resistance in ovarian cancer (9–11). The cellular DDR system detects and repairs damaged DNA to maintain a stable genome, and this inhibits cisplatin-mediated DNA damage (12, 13). These abovementioned three mechanisms of drug resistance in ovarian cancer have been studied in depth. However, the mechanism by which ncRNA causes drug resistance is unclear.

NcRNAs account for a majority of cellular RNAs and do not encode any functional proteins (14). After transcription, they mainly perform their biological functions at the RNA level (15). Unexpectedly, some ncRNAs promote drug resistance by inducing multiple cell phenotypes. Among the ncRNAs, long non-coding RNAs (lncRNAs), microRNAs (miRNAs), and circular RNAs (circRNAs) are thought to be mainly responsible for causing drug resistance in ovarian cancer. With a length of more than 200 nucleotides, lncRNAs regulate gene expression at the transcriptional, post-transcriptional, and epigenetic levels (16). They induce tumor cell stemness, apoptosis inhibition, abnormal tumor cell proliferation, drug efflux, protective autophagy, and epithelial-mesenchymal transition (EMT) to facilitate drug resistance in ovarian cancer. MiRNAs can be 20–25 nucleotides long (17). They recognize and bind mRNA by complementary base pairing, leading to mRNA degradation or translational inhibition (18). The resultant abnormally expressed mRNA can promote drug resistance by inducing apoptosis inhibition, abnormal glycolysis, drug efflux, and EMT in ovarian cancer. CircRNAs have a covalently closed loop structure and are relatively stable (19). They mainly act as competing endogenous RNAs (ceRNAs) to reverse the inhibitory effect of miRNAs on mRNA expression (20). Therefore, circRNAs regulate the expression of resistance-related proteins by regulating their mRNA expression, which promotes drug resistance in ovarian cancer.

This review summarizes the functional mechanisms and signaling pathways of lncRNAs, miRNAs, and circRNAs in ovarian cancer.

## Ectopic Expression of lncRNAs Mediates Chemotherapeutic Resistance

### Mechanisms of Action of lncRNAs

**Figure 1:**
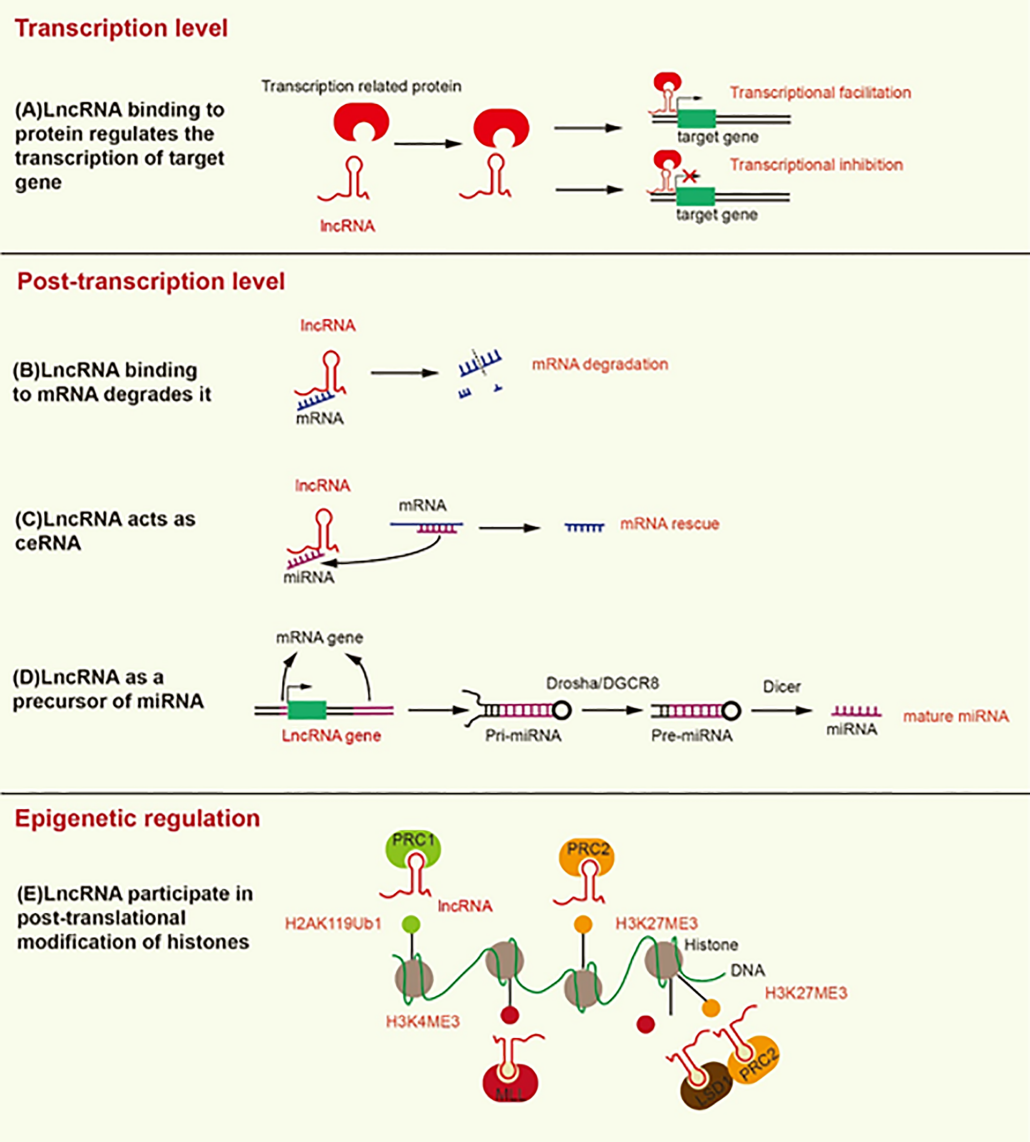
Mechanisms underlying the modification induced by lncRNA at the transcriptional, post-transcriptional, and epigenetic levels. **(A)** LncRNA binding to transcription-related proteins mediates transcriptional activation or transcriptional inhibition. **(B)** LncRNA promotes the degradation of mRNA by recruiting STAU1 to the SBS of dsRNA. **(C)** LncRNA, as a ceRNA, separates mRNA from its corresponding miRNA target. **(D)** LncRNA as a precursor of miRNA, develops into mature miRNA under the action of Drosha/DGCR8 and Dicer. **(E)** LncRNA contributes to the formation of H2AK119Ub1 and H3K27ME3, which inhibit gene transcription. In addition, lncRNA contributes to the formation of H3K4ME3, which promotes gene transcription.

At the transcriptional level, lncRNAs bind transcription factors to promote or inhibit the transcription of target genes (**Figure 1**). LncRNA HOTAIR recruits and binds the transcription factor SNAIL, which prevents it from binding to the hepatocyte nuclear factor 4 alpha (HNF4α) promoter, reducing its expression (21).

At the post-transcriptional level, the Alu element of lncRNA binds the Alu element of the 3’-untranslated region (3’UTR) in the target mRNA to form staufen1-binding site (SBS) by incomplete base-pairing. SBS binds Staufen1 (STAU1) to degrade the target mRNA (22). STAU1, as an RNA-binding protein, binds to SBS, leading to the degradation of mRNA in mammals (23). Therefore, lncRNA can directly interact with specific mRNA to degrade mRNA (**Figure 1**). On the contrary, as a type of ceRNA, lncRNA can sponge miRNA to release mRNA and restore the function of mRNA (**Figure 1**) (24). For example, LINC01118 sponges miR-134 to rescue the mRNA of ABCC1 (25). LncRNA also functions as a precursor of miRNA (**Figure 1**). Most miRNAs are derived from protein-coding genes in the human genome, but some miRNAs are derived from ncRNA-coding genes (26). Specifically, the genes encoding lncRNA are processed to form primary miRNA (pri-miRNA), and then pri-miRNA is further processed in the nucleus by Drosha/DGCR8 into a double-hairpin precursor miRNA (pre-miRNA) (27). Finally, the pre-miRNA is transported to the cytoplasm and cut into mature miRNA by Dicer (28). For instance, the exon of lncRNA H19 contains miR-675, and H19 acts as a precursor of miR-675 to regulate its expression (29). LncRNA can also change the cellular localization of proteins. For instance, lncRNA MALAT1 can bind YAP to inhibit its translocation from the nucleus to the cytoplasm (30).

LncRNA can mediate histone modification, resulting in epigenetic regulation (**Figure 1**). Both lncRNA ANRIL and lncRNA H19 can interact with enhancer of zeste 2 polycomb repressive complex 2 subunit (EZH2) and suppressor of zeste 12 (SUZ12) (31, 32). EZH2 and SUZ12 are subunits of polycomb repressive complex 2 (PRC2), and PRC2 inhibits the transcription of target genes by trimethylation at lysine 27 of histone H3 (H3K27Me3) (33). The interaction of ANRIL and H19 with EZH2 and SUZ12 can promote H3K27Me3 to inhibit the transcription of target genes (34, 35). In addition, lncRNA HOTTIP interacts with WD repeat domain 5 (WDR5) to promote trimethylation at lysine 4 of histone H3 (H3K4Me3), thereby promoting the transcription of target genes (36). WDR5 is a core component of mixed lineage leukemia (MLL), which catalyzes the formation of H3K4Me3 (36). LncRNA HOTAIR can interact with EZH2 and lysine specific demethylase 1 (LSD1), which is a histone demethylase that prevents the formation of H3K4Me3 to inhibit gene transcription. HOTAIR coordinates the interaction between EZH2 and LSD1 to detach the methyl groups from H3K4 to transform transcriptional activation into transcriptional inhibition of the target gene (37). Likewise, lncRNA ultraconserved element 338 (uc.338) binds BMI1, a subunit of PRC1, to monoubiquitinate histone H2A on lysine 119 (H2AK119ub1), which inhibits the transcription of target genes (38). We summarize the mechanisms and signaling pathways of lncRNAs that lead to platinum or taxane chemotherapeutic resistance in ovarian cancer (**Table 1**).

**Table 1:** Drug resistance in ovarian cancer caused by the ectopic expression of lncRNA.

Drug	LncRNA abbreviation	Pathway	Mode of action	Modes of drug resistance	Mechanism of resistance	References
Platinum	CCAT1	CCAT1/miR-454/survivin	ceRNA	Apoptosis inhibition	Upregulated CCAT1 induces apoptosis inhibition *via* miR-454/survivin pathway, resulting in drug resistance.	(39)	
	MALAT1	MALAT1/YAP	Protein translocation	Stemness	Upregulated MALAT1 promotes cell stemness *via* YAP, leading to drug resistance.	(30)	
	MALAT1	MALAT1/notch1/ABCC1	Protein expression	Drug efflux	Upregulated MALAT1 leads to drug efflux *via* notch1/ABCC1 pathway, which promotes resistance.	(40)	
	PANDAR	PANDAR/SFRS2/P53/P53-Ser15	Epigenetic regulation	Apoptosis inhibition	Upregulated PANDAR induces apoptosis inhibition *via* SFRS2/P53/P53-Ser15 pathway, leads to drug resistance.	(41)	
Taxane	LINC01118	LINC01118/miR-134/ABCC1	ceRNA	Drug efflux	Upregulated LINC01118 leads to drug efflux to promotes drug resistance *via* miR-134/ABCC1 pathway.	(25)	
	NEAT1	NEAT1/miR-194/ZEB1	ceRNA	EMT	Upregulated NEAT1 induces EMT to promote drug resistance *via* miR-194/ZEB1 pathway.	(42)	
	TUG1	TUG1/miR-29b-3p	ceRNA	Protective autophagy	Upregulated TUG1 induces protective autophagy leading to drug resistance *via* miR-29b-3p pathway.	(43)	
	UCA1	UCA1/miR-129/ABCB1	ceRNA	Drug efflux	Upregulated UCA1 leads to drug efflux to promote drug resistance *via* miR-129/ABCB1 pathway.	(44)	

ABCB1, ATP binding cassette subfamily B member 1; ABCC1, multidrug resistance-associated protein 1; EMT, epithelial mesenchymal transition; SFRS2, arginine/serine-rich 2; YAP, yes-associated protein; ZEB1, zinc finger E-box-binding homeobox 1.

### LncRNA-Mediated Chemotherapeutic Resistance Involves ABC Transporters

**Figure 2:**
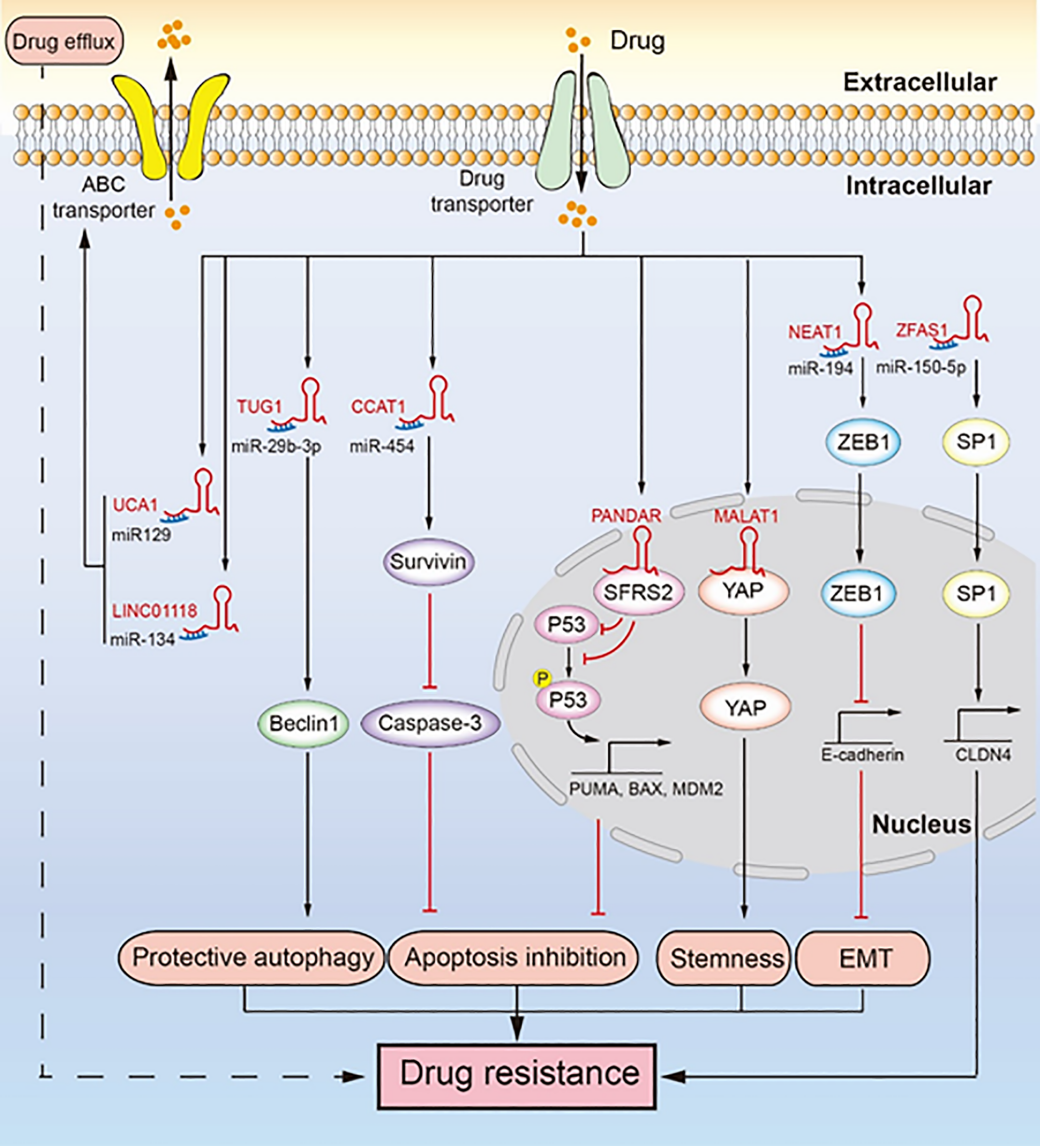
LncRNAs regulate chemotherapeutic resistance through diverse signaling pathways. Multiple lncRNAs promote drug resistance by inducing drug efflux, protective autophagy, apoptosis inhibition, tumor stemness, and epithelial-mesenchymal transition (EMT). LINC01118 and UCA1 regulate ABC transporters promoting drug resistance. TUG1 regulates Beclin1 leading to drug resistance induced by protective autophagy. CCAT1 upregulates survivin to inhibit the expression of caspase-3, which inhibits apoptosis and leads to drug resistance. PANDAR reduces the expression and phosphorylation of P53, inhibiting apoptosis and promoting drug resistance. MALAT1 binds YAP and inhibits its translocation from the nucleus to the cytoplasm, which facilitates cancer cell stemness leading to drug resistance. NEAT1 upregulates the expression of ZEB1, which promotes EMT leading to drug resistance. ZFAS1 promotes the transcription of CLDN4 to upregulate SP1, which leads to drug resistance.

ABC transporters are transmembrane proteins that can expel drugs from within cells through a process called drug efflux. LncRNAs promote the expression of some ABC transporters by sponging miRNA, which promotes drug efflux and induces drug resistance in ovarian cancer (**Figure 2**) (25, 40, 44). In ovarian cancer, both lncRNA MALAT1 and LINC01118 are upregulated, and they both promote drug efflux by increasing the expression of ABCC1 to induce drug resistance (25, 40). The difference is that MALAT1 can interact with the Notch1 protein to activate the Notch1 signaling pathway and promote the expression of ABCC1 to induce cisplatin resistance, while LINC01118 upregulates the expression of ABCC1 by sponging miR-134 to promote paclitaxel resistance. Moreover, the silencing of MALAT1 reduced tumor growth when ovarian tumor xenograft model mice were treated with cisplatin. Although lncRNA UCA1 promotes cisplatin resistance by inhibiting apoptosis (45), UCA1 can also promote paclitaxel resistance by inducing drug efflux through sponging of miR-129 to rescue ABCB1 expression (44). ABCB1 facilitates the elimination of chemotherapeutic drugs from cancer cells (46). UCA1 is highly upregulated in paclitaxel-resistant ovarian cancer cells. Drug efflux caused by ABC transporters is a very important drug resistance pathway. Given the lack of related studies, further studies are needed to understand how lncRNA makes ovarian cancer cells resistant to chemotherapeutic drugs through its action on ABC transporters. It will allow us to identify novel strategies to overcome the drug resistance of ovarian cancer.

### LncRNA-Mediated Chemotherapeutic Resistance Involves EMT

EMT involves transformation of epithelial cells, through loss of their polarity, into mesenchymal cells, which gives them the ability of invasion, migration, and anti-apoptosis (47). EMT induced by lncRNA promotes the malignancy of tumors and makes them resistant to chemotherapeutics (**Figure 2**) (42). For example, lncRNA NEAT1 is upregulated in paclitaxel-resistant ovarian cancer cells, which sponges miR-194 to restore the high expression of ZEB1. ZEB1 is essential for EMT because it inhibits the transcription of E-cadherin (48). In addition, NEAT1 knockdown significantly inhibited tumor growth in an ovarian tumor xenograft mouse model. EMT plays an important role in cancer metastasis and drug resistance, and the role of lncRNA-mediated EMT in the drug resistance of ovarian cancer needs further research.

### LncRNA Regulates Chemotherapeutic Resistance by Inducing Cancer Cell Stemness

Cancer cell stemness contributes to self-renewal and differentiation of cancer cells, which help tumor cells to regenerate and resist the toxicity of chemotherapeutic drugs (49). LncRNA can promote cancer cell stemness of ovarian cancer to promote drug resistance (**Figure 2**) (30). Specifically, MALAT1 is upregulated in cisplatin-resistant cells as well as in non-adherent spheres; it interacts with the YAP protein to inhibit its translocation to the cytoplasm from the nucleus, which leads to enhanced cancer cell stemness. While cancer cell stemness is a predominant mechanism contributing to drug resistance in ovarian cancer cells, not many studies have been conducted on cancer cell stemness induced by lncRNA. Therefore, there is further scope for research.

### LncRNAs Facilitate Chemotherapeutic Resistance by Promoting Abnormal Tumor Cell Proliferation

Abnormal cell proliferation in the presence of chemotherapeutic drugs indicates that the treatment is ineffective or that the cells have become resistant. LncRNA HOTAIR reduces the sensitivity of ovarian cancer to cisplatin by inducing abnormal tumor cell proliferation (50, 51). Mechanically, HOTAIR activates the Wnt/β-catenin signaling pathway to enhance the expression of cyclinD1 and CDK4. CyclinD1 is a marker of cell proliferation (50). It activates CDK4-expressing cells to progress from the G1 phase into the S phase, which accelerates cell cycle progression (52). In an ovarian tumor xenograft mouse model, HOTAIR downregulation inhibited tumor growth and cyclinD1 expression, and this inhibition effect was more remarkable when cisplatin was administered. The role of lncRNA HOTAIR in chemotherapeutic resistance caused by the abnormal proliferation of ovarian cancer cells and other tumor cells is understudied, necessitating further studies.

### LncRNA-Mediated Chemotherapeutic Resistance Involves Induction of Protective Autophagy

Protective autophagy is another mechanism contributing to chemotherapeutic resistance (53). Specifically, cancer cells undergo protective autophagy to obtain nutrients and promote their survival by degrading metabolic waste, damaged proteins, and damaged organelles, thereby increasing their resistance against chemotherapeutic drugs. LncRNA TUG1 mediates paclitaxel resistance by inducing protective autophagy in ovarian cancer (**Figure 2**) (43). Specifically, TUG1 is upregulated in cisplatin-resistant cells and sponges miR-29b-3p to indirectly upregulate the expression of Beclin1, which increases autophagosome formation in ovarian cancer (43). In an ovarian tumor xenograft mouse model, TUG1 promoted tumor growth by resisting the effect of paclitaxel, and downregulation of TUG1 decreased the tumor size and weight. Drug resistance due to protective autophagy in tumors is a hot topic of current research, and the role of lncRNA in this mechanism has gained attention. Although autophagy has long been known to contribute to drug resistance in cancer cells, the ability of lncRNA to induce protective autophagy and resulting in ovarian cancer drug resistance seems to be a new research direction.

### LncRNAs Promote Chemotherapeutic Resistance by Inhibiting Apoptosis

Apoptosis is the self-destructive mechanism of cells, and apoptosis inhibition rescues cancer cells and induces chemotherapeutic resistance. LncRNAs regulate apoptosis-related proteins to induce apoptosis inhibition, thereby inducing cisplatin resistance in ovarian cancer (**Figure 2**) (39, 41, 45). Mechanistically, lncRNA CCAT1 is upregulated in cisplatin-resistant ovarian cancer cells, which enhances the expression of survivin as lncRNA CCAT1 sponges miR-454 (39). Survivin, an inhibitor of apoptosis, can bind and inhibit caspase-9, caspase-3, and caspase-7, which hinder apoptosis and cause cisplatin resistance (54). In the nucleus, lncRNA PANDAR binding the SFRS2 protein downregulates the expression of P53 and its phosphorylation at serine 15 (Ser-15), which inhibits the transcription of P53-mediated pro-apoptotic genes, including MDM2, BAX, and PUMA (41). In addition, PANDAR is upregulated by cisplatin. Likewise, lncRNA UCA1 induces cisplatin resistance by indirectly promoting the expression of SPRK1 and BCL-2 and inhibiting the expression of BAX, caspase-3, and caspase-9 (45). LncRNA NEAT1 regulates miR-770-5p/PARP1 signaling to induce cisplatin resistance in ovarian cancer (55). NEAT1 is overexpressed in cisplatin-resistant ovarian cancer cells and sponges miR-770-5P to upregulate the expression of poly adenosine diphosphate-ribose polymerase 1 (PARP1), which leads to chemotherapeutic resistance in cancer (56). Recent studies have shown that lncRNA SNHG22 is highly expressed in ovarian cancer tissues and promotes cisplatin resistance (57). SNHG22 sponges miR-2467 to enhance Gal-1 expression, and Gal-1 activates the H-Ras/Raf/ERK pathway to induce apoptosis inhibition and drug resistance (58). Likewise, lncRNA EPEI upregulation indirectly downregulates P53 expression, leading to carboplatin resistance in ovarian endometrioid adenocarcinoma (59). Inactivation of P53, a tumor suppressor gene, can promote carcinogenesis and inhibit cell apoptosis (60). LncRNA NCK adaptor protein 1 (NCK1)-AS1 not only sponges miR-137 to upregulate NCK1 expression but also directly interacts with c-CBI to inhibit its degradation caused by ubiquitination (61). This inhibits the apoptosis of ovarian cancer cells and makes them resistant to cisplatin (61, 62). In an ovarian tumor xenograft mouse model, the knockdown of CCAT1 reduced tumor weight, whereas PANDAR overexpression increased tumor volume (39, 41). In addition, P53 and PUMA were downregulated in a PANDAR-overexpressing xenograft mouse model. In ovarian cancer apoptosis inhibition is a well-known drug resistance mechanism. Here, lncRNA does not directly regulate the classic apoptosis pathway to induce drug resistance caused by apoptosis inhibition. Therefore, in-depth study of lncRNA may provide new insights into the classic apoptosis pathway.

### LncRNAs Induce Chemotherapeutic Resistance by Mediating DNA Regulatory Proteins

LncRNAs induce chemotherapeutic resistance of ovarian cancer by acting on special DNA regulatory proteins (57, 63–67). LncRNA HOTAIR can facilitate the expression of Homeobox A7 (HOXA7) and sponge miR-138-5p to rescue EZH2 and sirtuin 1 (SIRT1) expression in ovarian cancer, both of which contribute to cisplatin resistance (63, 68). EZH2 is involved in histone methylation, while SIRT1 mediates histone deacetylation (69, 70). In addition, HOTAIR influences the DNA damage response to promote cisplatin resistance in ovarian cancer (64). ANRIL overexpression confers makes ovarian cancer cells resistant to cisplatin through the let-7a/high-mobility group protein A2 (HMGA2) axis (65). HMGA2 can also influence the proliferation and differentiation of cells by upregulating PRC2 (71–73). LncRNA ZFAS1 is overexpressed in epithelial ovarian cancer cells and directly targets miR-150-5p to enhance the expression of specificity protein 1 (SP1), which makes ovarian cancer cells resist to cisplatin and paclitaxel (66). Another study showed that SP1, as a transcription factor, facilitates the transcription of claudin-4 (CLDN4), which causes low DNA methylation and high histone H3 acetylation in the CLDN4 promoter region (74, 75). In an ovarian tumor xenograft mouse model, downregulation of HOTAIR and ANRIL could slow down tumor growth (63, 65). In addition, HOTAIR downregulation decreased the protein levels of HOXA7 *in vivo*. Understanding the role of DNA regulation in organisms is still a difficult problem, and research on lncRNA-mediated DNA regulatory proteins to promote drug resistance remains at a relatively superficial level, which requires more researches to reveal it.

## Ectopic Expression of miRNAs Promotes Chemotherapeutic Resistance in Ovarian Cancer

### Mechanisms of Action of miRNAs

MiRNA is a type of endogenous ncRNA with regulatory functions (76). It is processed by nucleases from longer primary transcripts and is 20-25 nucleotides long. MiRNA mainly forms a silencing complex, which binds to the 3’UTR region of the target mRNA through complementary base pairing and controls the stability and translation of the mRNA (18). The specific mechanism of miRNA-induced drug resistance in ovarian cancer is described below. Here, we summarize the mechanisms and signaling pathways of miRNAs that lead to platinum or taxane chemotherapeutic resistance in ovarian cancer (**Table 2**).

**Table 2:** Drug resistance in ovarian cancer caused by the ectopic expression of miRNA and circRNA.

Drug	RNA Abbreviation	Pathway	Mode of action	Modes of drug resistance	Mechanism of resistance	References
Platinum	miR-1180	SFRP1/Wnt-5a/β-catenin	Inhibition of mRNA	Glycolysis	Upregulated miR-1180 promotes glycolysis to induce drug resistance *via* SFRP1/Wnt-5a/β-catenin pathway.	(77)
	miR-223	PTEN/PI3K/AKT	Inhibition of mRNA	Apoptosis inhibition	Upregulated miR-223 induces apoptosis inhibition to promote drug resistance *via* PTEN/PI3K/AKT pathway.	(78)
	miR-149-5p	MST1, SAV1/YAP, TAZ	Inhibition of mRNA	Apoptosis inhibition	Upregulated miR-149-5p induces apoptosis inhibition to lead to drug resistance *via* MST1, SAV1/YAP, TAZ pathways.	(79)
	miR-106a	PDCD4/caspase-3, caspase-8	Inhibition of mRNA	Apoptosis inhibition	Upregulated miR-106a inhibits apoptosis to promote drug resistance *via* PDCD4/caspase-3, caspase-8 pathway.	(80)
	miR-93	PTEN/AKT	Inhibition of mRNA	Apoptosis inhibition	Upregulated miR-93 inhibits apoptosis to promote drug resistance *via* PTEN/AKT pathway.	(81)
	miR-214	PTEN/AKT	Inhibition of mRNA	Apoptosis inhibition	Upregulated miR-214 inhibits apoptosis to promote drug resistance *via* PTEN/AKT pathway.	(82)
	miR-205	PTEN	Inhibition of mRNA	Apoptosis inhibition	Upregulated miR-205 induces apoptosis inhibition to promote drug resistance *via* the PTEN pathway.	(83)
	miR-411	ABCG2	Inhibition of mRNA	Drug efflux	Downregulated miR-411 leads to drug efflux to induce to drug resistance *via* the ABCG2 pathway.	(84)
	miR-142-5p	MCL-1	Inhibition of mRNA	Apoptosis inhibition	Downregulated miR-142-5p inhibits apoptosis to promote drug resistance *via* the MCL-1 pathway.	(85)
	miR-204	IL-6R/STAT3/miR-204/IL-6R	Inhibition of mRNA	Apoptosis inhibition	Downregulated miR-204 in IL-6R/STAT3/miR-204/IL-6R pathway induces apoptosis inhibition to promote drug resistance.	(86)
	miR-125b	BAK1	Inhibition of mRNA	Apoptosis inhibition	Upregulated miR-125b induces apoptosis inhibition to promote drug resistance *via* the BAK1 pathway.	(87)
	miR-93	PTEN/AKT	Inhibition of mRNA	Apoptosis inhibition	Upregulation of AKT leads to drug resistance.	(88)
	miR-216a	PTEN	Inhibition of mRNA	Apoptosis inhibition	Downregulation of PTEN leads to drug resistance.	(89)
	miR-21	PTEN	Inhibition of mRNA	Apoptosis inhibition	Downregulation of PTEN leads to drug resistance.	(90)
Taxane	miR-1307	CIC/ETV4	Inhibition of mRNA	Drug efflux	Upregulated miR-1307 may lead to drug resistance by promoting MDR1 transcription *via* CIC/ETV4 pathway.	(91)
	miR-630	APAF-1	Inhibition of mRNA	Apoptosis inhibition	Upregulated miR-630 inhibits apoptosis leading to drug resistance *via* the APAF-1 pathway.	(92)
	miR-106a	Caspase-7; BCL10	Inhibition of mRNA	Apoptosis inhibition	Upregulated miR-106a inhibits apoptosis leading to drug resistance *via* caspase-7, BCL10 pathway.	(93)
	miR-29b	BAG3/miR-29b/MCL-1	Inhibition of mRNA	Apoptosis inhibition	Downregulated miR-29b in BAG3/miR-29b/MCL-1 pathway induces apoptosis inhibition to promote drug resistance.	(94)
	miR-27a	HIPK2/MDR1/P-gp	ceRNA	Drug efflux	Downregulation of HIPK2 promotes the transcription of MDR1, thus promoting drug resistance.	(95)

ABCG2, ATP-binding cassette transporter G2; AKT, protein kinase B; APAF-1, apoptotic protease activating factor-1; BAK1, BCL2Antagonist/Killer 1; CIC, capicua transcriptional repressor; ETV4, ETS Variant Transcription Factor 4; HOXC8, homeobox C8; IL-6R, interleukin-6 receptor; BAG3, Bcl2-associated athanogene 3; Mcl-1, myeloid cell leukemia 1; MD1R, multi-drug resistance-1; MST1, macrophage stimulating 1; PDCD4, programmed cell death 4; PI3K, phosphatidylinositide 3-kinases; PTEN, phosphatase and tensin homolog; SAV1, salvador homolog 1; SFRP1, secreted frizzled-related protein 1; STAT3, signal transducer and activator of transcription 3; TAZ, tafazzin; YAP, yes-associated protein.

### MiRNA-Induced Chemotherapeutic Resistance Involves ABC Transporters

Multiple miRNAs have been found to regulate the expression of ABC transporters to mediate drug efflux, leading to chemotherapeutic resistance (84, 91, 95, 96). Specifically, miR-130a, miR-1307, and miR-27a promote drug resistance in ovarian cancer by increasing the expression of P-glycoprotein (P-gp) (91, 95, 96). P-gp, a drug transporter encoded by multi-drug resistance-1 (MDR1), is also called ABCB1. It promotes drug resistance through its drug efflux function. MiR-130a is overexpressed in cisplatin-resistant ovarian cancer cells, and it indirectly enhances the expression of P-gp. MiR-1307 and miR-27a are highly expressed in paclitaxel-resistant ovarian cancer cells. Mechanically, miR-1307 relieves the transcriptional repression of ETV4 by directly downregulating CIC expression, and ETV4 upregulates the transcription of MDR1 by binding to the MDR1 promoter region (97, 98). MiR-27a targets homeodomain-interacting protein kinase-2 (HIPK2), which reduces the transcriptional repression of MDR1 caused by HIPK2 (99). Downregulation of miR-411, mediated by low levels of SLC27A2, enhances the expression of ABCG2, which promotes drug efflux to induce cisplatin resistance in ovarian cancer (84). The common members of the ABC transporter family are ABCC1, ABCB1, and ABCG2. ABCC1 and ABCG2 are less frequently reported in studies related to miRNA and ABC transporters. Therefore, more studies on the miRNA-induced ABCB1 or ABCG2 expression, which leads to drug resistance in ovarian cancer, will improve our understanding of the drug resistance mechanism of ABC transporters.

### MiRNA-Mediated Chemotherapeutic Resistance Involves EMT

EMT has been widely implicated in the malignant behavior of tumors. It promotes tumor invasion and migration. Li et al. (100) found that miR-181a is overexpressed in paclitaxel-resistant ovarian cancer cells, and it induces paclitaxel resistance by facilitating EMT through the upregulation of N-cadherin and downregulation of E-cadherin. N-cadherin is a positive regulator, and E-cadherin is a negative regulator of EMT (101). The malignant phenotype induced by miRNA-induced EMT may be the key to ovarian cancer drug resistance, which requires further research.

### MiRNA-Mediated Chemotherapeutic Resistance Involves Upregulation of Glycolysis

**Figure 3:**
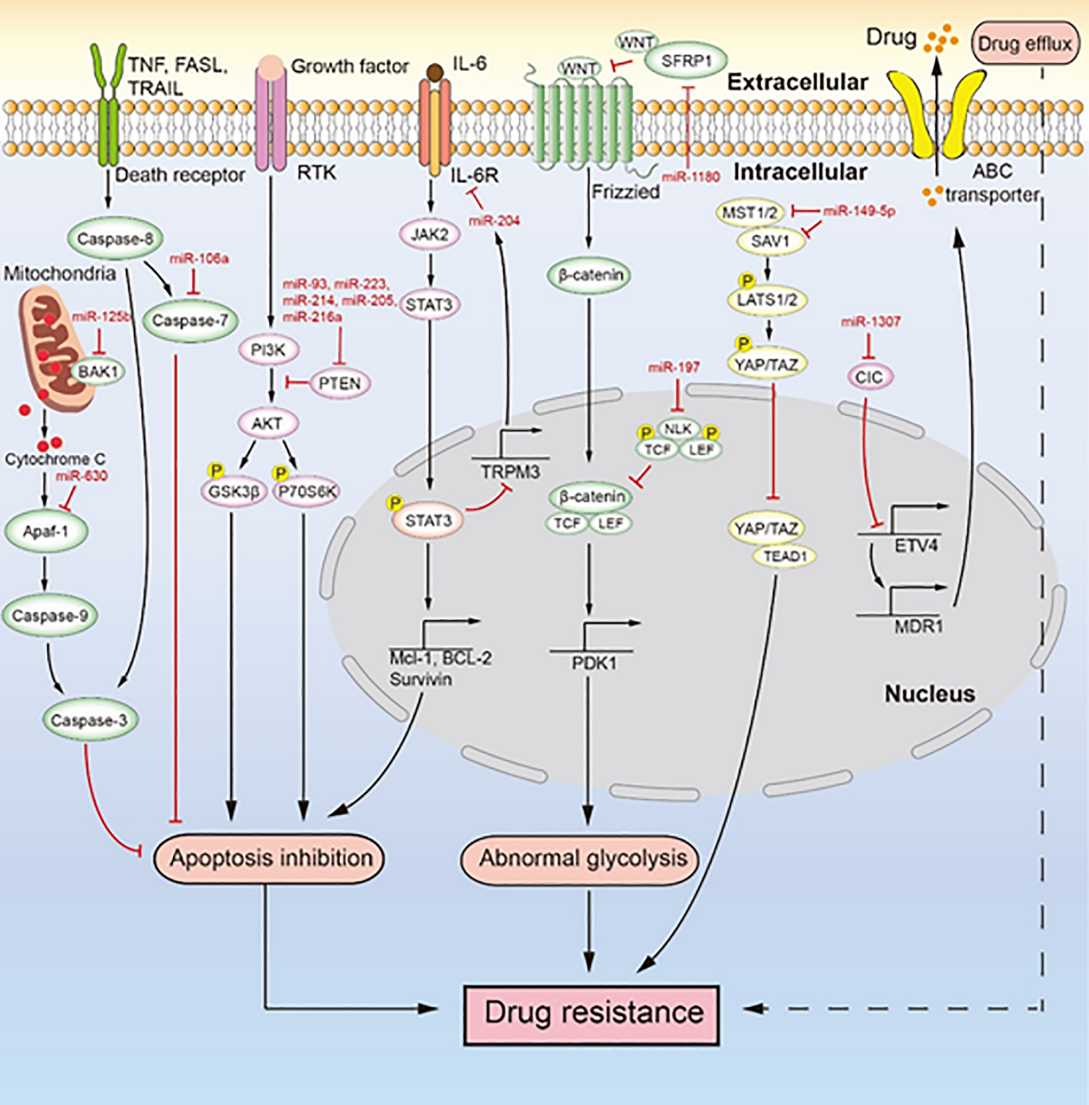
MiRNAs mediate chemotherapeutic resistance by multiple signaling pathways. Multiple miRNAs lead to drug resistance by inducing drug efflux, apoptosis inhibition, and abnormal glycolysis. MiR-106a, miR-630, and miR-125b inhibit apoptosis to induce drug resistance. MiR-93, miR-223, miR-214, miR-205, and miR-216a regulate the PI3K/AKT signaling pathway to inhibit apoptosis, which induces drug resistance. The IL-6/STAT3/miR-204 feedback loop inhibits apoptosis to promote drug resistance. MiR-1180 and miR-197 act *via* the Wnt signaling pathway to promote drug resistance. MiR-149-5p regulates the expression of MST1/2 and SAV1 to facilitate drug resistance. MiR-1307 upregulates the expression of ABCB1 to promote drug resistance.

Glycolysis is beneficial for the malignant phenotype of tumors as it provides energy for the metabolism of ovarian cancer cells to induce cisplatin resistance (**Figure 3**) (77, 102). MiR-1180 induces the abnormal upregulation of glycolysis by activating the Wnt signaling pathway and its downstream components including Wnt5a, β-catenin, c-Myc, and CyclinD1 proteins that can enhance glycolysis. MiR-1180 targets SFRP1 to relieve its inhibitory effect on Wnt5a, which activates the Wnt/β-catenin signaling pathway to upregulate PDK1 expression. PDK1 is a key enzyme required for the glycolysis of tumor cells and is transcribed by the combination of lymphoid enhancer factor/T-cell factor (LEF/TCF) and β-catenin (103). This abnormal upregulation of glycolysis is a cause of cisplatin resistance in ovarian cancer. Drug resistance in ovarian cancer cells induced by miRNA-mediated abnormal glycolysis is not extensively studied; therefore, further research on this topic may reveal a new link between miRNA and drug resistance.

### MiRNAs Regulate Chemotherapeutic Resistance by Inhibiting Apoptosis

As the main reason for chemotherapeutic resistance, apoptosis inhibition is induced by the abnormal expression of miRNAs to cause chemotherapeutic resistance in ovarian cancer (**Figure 3**). For instance, Xu et al. (79) reported that miR-149-5p is highly expressed in cisplatin-resistant ovarian cancer cells, and inactivation of the Hippo signaling pathway induces cisplatin resistance by directly inhibiting the expression of MST1 and SAV1. The downregulation of MST1 and SAV1 reduces the phosphorylation of YAP and TAZ through inhibition of the phosphorylation of LAST1/2. This enhances the nuclear levels of YAP and TAZ, and their upregulation inhibits the activity of caspase-3 and caspase-9, leading to apoptosis inhibition. MiR-106a is highly expressed in cisplatin-resistant and paclitaxel-resistant ovarian cancer cells (80, 93). It not only targets PDCD4 to downregulate the level of cleaved caspase-8 and cleaved caspase-3 in the death receptor pathway but also directly inhibits the expression of caspase-7 and BCL10. Therefore, miR-106a inhibits cell apoptosis by downregulating the expression of apoptosis-related proteins and makes ovarian cancer cells resistant to cisplatin and paclitaxel. MiR-214 targets PTEN to induce cisplatin resistance in ovarian cancer (82). The overexpression of miR-214 in ovarian cancer and the inhibition of PTEN expression reduce the activation of the AKT pathway, which promotes the phosphorylation of glycogen synthase kinase 3h (GSK3β) and p70 Ribosomal Protein S6 Kinase (p70S6K). Thus, miR-214 facilitates apoptosis inhibition to induce cisplatin resistance. Similarly, the upregulation of miR-93, miR-223, or miR-216a expression can promote the cisplatin resistance of ovarian cancer through activation of the PI3K/AKT pathway caused by the sponging of PTEN (78, 81, 88, 89, 104). In addition, miR-21, miR-130a, miR-205, and miR-93 can negatively regulate the expression of PTEN, which makes ovarian cancer cells resistant to cisplatin (83, 90, 96, 105). MiR-125b is highly expressed in cisplatin-resistant ovarian cancer cells and targets BAK1 to induce cisplatin resistance (87). As a pro-apoptotic protein, BAK1 enhances the mitochondrial permeability and promotes the release of cytochrome C, thereby promoting the occurrence of mitochondrial apoptosis (106). PRKCD is considered to induce apoptosis (107). MiR-204, as a key factor of the IL-6R/STAT3/miR-204 feedback loop, causes cisplatin resistance in epithelial ovarian cancer (86). Mechanistically, the binding of interleukin-6(IL-6) to IL-6R activates JAK2 to promote the level and nuclear translocation of p-STAT3, which enhances the transcription of anti-apoptotic proteins (MCL-1, BCL-2, and survivin). It also binds to the promoter region of TRPM3 to inhibit the transcription of miR-204, which the then enhances the level of its target protein IL-6R, further activating the transcription of anti-apoptotic proteins induced by p-STAT3 to facilitate cisplatin resistance. The high expression of miR-630 in paclitaxel-resistant ovarian cancer cells induces apoptosis inhibition by directly downregulating APAF-1 (92, 108). As an activator of mitochondrial apoptosis, APAF-1 induces apoptosis in ovarian cancer. In an ovarian tumor xenograft mouse model, the upregulation of miR-223 and miR-205 decreased tumor growth and the expression of PTEN (78, 83). Likewise, the high expression of miR-204 enhanced cisplatin resistance, and the low expression of miR-630 increased paclitaxel sensitivity in an ovarian tumor xenograft mouse model (86, 92). Both miR-204 and miR-630 promote the malignant phenotype of tumor cells. MiRNA inhibits the apoptosis of ovarian cancer cells mainly through inhibition of the death receptor pathway, inhibition of the mitochondrial apoptosis pathway, and activation of the PI3K/AKT pathway. These three signaling pathways are extensively studied, but the role of miRNA in these pathways seems like an unexplored area of research. Studies in this area will deepen our understanding of the drug resistance of ovarian cancer caused by apoptosis inhibition.

## Ectopic Expression of circRNAs Induces Chemotherapeutic Resistance

CircRNA is a new research hotspot in the regulation of drug resistance of ovarian cancer by ncRNAs, but research on this topic is still in its nascent stages (109–112). Luo et al. (109) found that circFoxp1 upregulates the level of CCAAT enhancer binding protein gamma (CEBPG) and formin-like 3 (FMNL3) to promote cisplatin resistance by sponging miR-22 or miR-150-3p. CEBPG and FMNL3, two oncogenes in ovarian cancer, are common targets of miR-22 and miR-150-3p, respectively. Similarly, circTNPO3, circCELSR1, and circNRIP1 are highly expressed in paclitaxel-resistant ovarian cancer cells (110–112). CircTNPO3 increases the expression of NIMA-related kinase 2 (NEK2) by directly targeting miR-1299, which then contributes to chemotherapeutic resistance in ovarian cancer (113). CircCELSR1 regulates the miR-1252/forkhead box R2 (FOXR2) axis to facilitate paclitaxel resistance in ovarian cancer. CircNRIP1 upregulates the expression of HOXC8 by sponging miR-211-5p, which makes ovarian cancer cells less sensitive to paclitaxel. HOXC8 enhances the expression of PCNA, CyclinD1, Bcl-2, MMP2, and MMP9, thereby inhibiting the expression of cleaved caspase-3. In an ovarian tumor xenograft mouse model, the knockdown of circTNPO3, circCELSR1, or circNRIP1 slowed down tumor growth (110–112). In addition, the knockdown of circTNPO3 or circNRIP1 increased paclitaxel sensitivity *in vivo*. In recent years, research on circRNA has been gaining momentum, but there is a lot of scope for further research. Therefore, future research could focus on the relationship between circRNA and ovarian cancer resistance, which will open avenues for targeting ncRNAs to overcome drug resistance in ovarian cancer.

## Conclusions

Many types of ncRNAs exist, and each type has many different members. In this review, we have covered most members of lncRNA, miRNA, and circRNA families that have been found to be associated with drug resistance in ovarian cancer in recent years and their mechanisms leading to drug resistance. Studies on ncRNAs in ovarian cancer drug resistance have mainly focused on the well-known drug resistance pathways of ncRNA, such as apoptosis inhibition, drug efflux, and EMT. Through these studies, we can understand the role of ncRNAs in tumors, their function at the molecular level, and their response to chemotherapeutic drugs. Additional in-depth studies on these ncRNAs in terms of the signaling pathways that they are involved in, including identifying the specific upstream and downstream factors, will help us understand how ncRNAs induce ovarian cancer drug resistance and lead to poor prognosis. However, the main challenge is to screen and isolate the most prominent ncRNAs related to drug resistance from a large pool of ncRNAs.

## Future Perspectives

Systemic chemotherapy is currently the main treatment strategy for ovarian cancer patients. Unfortunately, drug resistance remains an inevitable problem in the long-term chemotherapy of cancer. Interventions aimed at abnormally expressed ncRNAs have shown promise in reversing the drug resistance of ovarian cancer. Currently, several ways have been proposed to achieve ncRNA-targeted therapy including the upregulation ofncRNAs by utilizing mimics, the exogenous expression or downregulation of ncRNAs by using small interfering RNAs (siRNAs) or short hairpin RNAs (shRNAs), and inhibition of ncRNA function through antisense oligonucleotides. These methods will allow us to regulate the expression of ncRNAs restore the drug sensitivity of drug-resistant ovarian cancer cells. Therefore, the combination of ncRNA-targeted therapy and chemotherapy may be a promising method for the treatment of ovarian cancer in the future. However, it is a major challenge to accurately and effectively apply these ncRNA modulators to the human body. It is reported that incorporation of specific oligonucleotides into nanoparticles can improve their delivery efficiency, thereby achieving optimal therapeutic effect on tumors (114). In addition, GalNAc-siRNA conjugates have been shown to accurately act as siRNAs against mRNAs in cells; therefore, this technology can be explored to counter ovarian cancer drug resistance (115). However, prior to the clinical application of such technology, research on its safety and practicality is indispensable. More relevant clinical trials are required to understand the true clinical potential of the above-mentioned ncRNAs, so that we can develop novel and effective treatment strategies for ovarian cancer.

## Author Contributions 

GL, JG, SC, ZW, and HZ: contributed to this article with the design. GL, JG, ZW, DC, JZ, XH, and JT: literature search. GL, JG, SC, YY, and WC: drafting. GL, JG, SC, ZW, DC, WC, and HZ: revision. GL, JG, ZW, JZ, XH, JT, WC, and HZ: editing. HZ: final approval. All authors contributed to the article and approved the submitted version.

## Funding

Our work was supported by the Key Project of Joint Funds of Hubei Health and Family Planning Commission (Grant No. WJ2018H174), the Postgraduate Innovation Fund Project of Yangtze University Health Science Center (Grant No. 202006), and the Natural Science Foundation of Hubei Province (Grant No. 2017CFB703).

## Conflict of Interest

The authors declare that the research was conducted in the absence of any commercial or financial relationships that could be construed as a potential conflict of interest.

## Publisher’s Note

All claims expressed in this article are solely those of the authors and do not necessarily represent those of their affiliated organizations, or those of the publisher, the editors and the reviewers. Any product that may be evaluated in this article, or claim that may be made by its manufacturer, is not guaranteed or endorsed by the publisher.
